# Potential effects of acanthocephalan and microsporidian parasites on the trophic status of the freshwater isopod *Asellus aquaticus*

**DOI:** 10.1051/parasite/2025063

**Published:** 2025-11-17

**Authors:** Annemie Doliwa, Michelle Musiol, Milen Nachev, Daniel Grabner, Willem Kaijser, Bernd Sures

**Affiliations:** 1 Aquatic Ecology, University of Duisburg-Essen 45141 Essen Germany; 2 Centre for Water and Environmental Research (ZWU), University of Duisburg-Essen 45141 Essen Germany; 3 Research Center One Health Ruhr, Research Alliance Ruhr, University of Duisburg-Essen 45141 Essen Germany

**Keywords:** Diversity, Host-parasite interactions, Stable Isotope Analysis, Trophic ecology

## Abstract

Parasites are known for their ability to induce a variety of changes in their respective hosts, including morphological characteristics and trophic interactions. For many host-parasite relationships, however, these aspects are yet to be explored. We assessed the occurrence of acanthocephalans and microsporidians in a population of the isopod *Asellus aquaticus* from a stream in western Germany over several months. We aimed to contrast the trophic positions of Acanthocephala-infected, Microsporidia-infected and uninfected isopods by assessing the stable isotope ratios for nitrogen (δ^15^N) and carbon (δ^13^C). We found acanthocephalans of the genus *Acanthocephalus* as well as five different microsporidian species, three of which are novel isolates. Prevalences were generally low among the 538 tested isopods (1.3% in September to 4.0% in January for acanthocephalans, and 0.7% in January to 12.3% in November for microsporidians), with a strong peak of microsporidian infections in November. The stable isotope analysis revealed temporal shifts in both δ^13^C and δ^15^N values, probably corresponding to dietary changes. Isopods infected with the microsporidian isolate EFB02 were enriched in ^15^N compared to uninfected ones, suggesting possible infection-associated physiological or metabolic changes. Acanthocephalan-infected isopods resembled uninfected ones in the two autumn samplings, but showed elevated δ^15^N values in September and January. This pattern may reflect active development of cystacanths in September and January, possibly linked to higher nutrient demands. Our findings emphasize the ecological importance of parasite infections in freshwater detritivores and underscore the need to consider the environmental and temporal context in host-parasite trophic studies.

## Abstract (fr)

Les parasites sont connus pour leur capacité à induire divers changements chez leurs hôtes respectifs, notamment au niveau des caractéristiques morphologiques et des interactions trophiques. Cependant, pour de nombreuses relations hôtes-parasites, ces aspects restent à explorer. Nous avons évalué la présence d’Acanthocéphales et de Microsporidies dans une population d’isopodes, *Asellus aquaticus*, d’un ruisseau de l’ouest de l’Allemagne pendant plusieurs mois. Notre objectif était de comparer les positions trophiques des isopodes infectés par des Acanthocéphales, infectés par des Microsporidies et non infectés en évaluant les rapports isotopiques stables de l’azote (δ^15^N) et du carbone (δ^13^C). Nous avons identifié des Acanthocéphales du genre *Acanthocephalus* ainsi que cinq espèces différentes de Microsporidies, dont trois sont de nouveaux isolats. Les prévalences étaient généralement faibles parmi les 538 isopodes testés (1,3 % en septembre à 4,0 % en janvier pour les Acanthocéphales, et 0,7 % en janvier à 12,3 % en novembre pour les Microsporidies) avec un fort pic d’infections par les Microsporidies en novembre. L’analyse des isotopes stables a révélé des décalages temporels dans les valeurs de δ^13^C et δ^15^N, correspondant probablement à des changements alimentaires. Les isopodes infectés par l’isolat de Microsporidies EFB02 étaient enrichis en ^15^N par rapport aux isopodes non infectés, suggérant de possibles changements physiologiques ou métaboliques associés à l’infection. Les isopodes infectés par les Acanthocéphales ressemblaient à ceux non infectés dans les deux échantillonnages d’automne, mais ont montré des valeurs de δ^15^N élevées en septembre et janvier. Ce schéma pourrait refléter un développement actif des cystacanthes en septembre et janvier, possiblement lié à des besoins nutritionnels plus élevés. Nos résultats soulignent l’importance écologique des infections parasitaires chez les détritivores d’eau douce et soulignent la nécessité de prendre en compte le contexte environnemental et temporel dans les études trophiques hôte-parasite.

## Introduction

Parasites play an influential role in food webs and thus in ecosystem functioning [32]. For example, they can modify their host’s behavior, growth or morphology, and by doing so, they can alter trophic interactions and cascades [30, 62]. For many ecologically important aquatic organisms, the effects of parasite infections remain poorly understood [60], and the identity and diversity of their associated parasites are often inadequately documented. The knowledge gaps extend to keystone species such as *Asellus aquaticus*, a widespread freshwater isopod in Europe. As a resilient detritivore, *A. aquaticus* inhabits a broad range of habitats, including fresh to brackish waters with slow-flowing or stagnant conditions [31, 51, 59].

*Asellus aquaticus* serves as a host for two phylogenetically distinct groups of obligate parasites: the multicellular Acanthocephala, as well as the unicellular Microsporidia. Acanthocephala are heteroxenous parasites that are trophically transmitted from an arthropod intermediate to a vertebrate final host [42, 61]. Several species of the genus *Acanthocephalus* employ *A. aquaticus* as their intermediate host and reside in its haemocoel, like the larval stages of *Acanthocephalus lucii* and *Acanthocephalus anguillae*, both of which parasitize the intestines of freshwater fish as adults [5, 10]. *Acanthocephalus* species can induce morphological changes in isopods, such as altering the body size [24, 27], pigmentation [41, 57], and sexual maturation [27]. Behavioral changes raising the predation risk of *A. aquaticus* for the acanthocephalan to reach its final host are also reported [3]. In contrast to these comparably large cystacanths, Microsporidia are obligate intracellular parasites that exhibit two primary modes of transmission: horizontal transmission *via* environmentally resistant spores released from infected hosts, and vertical transmission through the ovaries and eggs of infected females, thereby passing directly to the offspring [21]. The relationships of Microsporidia with *A. aquaticus* are only poorly understood: the only formally described species in *A. aquaticus* so far is *Mrazekia argoisi* which infects fat body cells of its host [29]. Nearly a hundred years after this species description, however, a Europe-wide barcoding study revealed broad diversity of microsporidian isolates in *A. aquaticus* [20]. The nature of a microsporidian infection can strongly differ depending on the species, including development in different tissues and infection intensities. Thus, a broad variety of effects has been observed in crustaceans, ranging from altered behavior [1], altered body size [20], and excess host feminization [26] to increased mortality [11]. Although *A. aquaticus* infected with microsporidians often show increased body size [20], the broader physiological and metabolic effects of such infections remain poorly understood. Given the distinct life cycles and exploitation strategies of acanthocephalans and microsporidians, their nutritional demands and impacts on host metabolism are likely to differ, though these differences have yet to be elucidated.

A valuable tool in ecology to explore interactions between organisms in a trophic context is stable isotope analyses (SIA) of carbon (^13^C) and nitrogen (^15^N). Studies on the stable isotopes (SIs) of carbon and nitrogen have shown that consumers exhibit enrichment of approximately 3.4‰ in δ^15^N (the ratio of ^15^N to ¹⁴N) and 0–1‰ in δ^13^C (the ratio of ^13^C to ^12^C) with each trophic transfer relative to their diet [8, 34]. Accordingly, isotopic discrimination – the difference in isotopic signatures between predator and prey or consumer and diet – serves as a distinctive fingerprint that allows us to determine an organism’s food sources and to understand their trophic interactions. However, the majority of SIA-based studies are focused on predator-prey or herbivore-plant relationships, whereas parasite-host trophic interactions have less frequently been studied. Available studies on different host-parasite systems showed that parasites do not always follow the consumer-diet fractionation patterns found for free-living organisms [38]. For example, adult endoparasites like Acanthocephala and Cestoda can be depleted in ^15^N with respect to their definite host [18, 43], while ectoparasites can be enriched in ^15^N compared to their hosts [9, 44]. Parasites can also induce changes in the host’s isotopic signatures (summarized in [4]), as was observed for *Daphnia* infected with Microsporidia, leading to enrichment in δ^13^C and δ^15^N [52]. Such altered signatures can, for example, be a result of changes in the diet composition or of host starvation [4]. In this context, *A. aquaticus* is an excellent target organism for such SI studies, as it is host to two very distinct parasite groups and thus allows direct comparisons between possible alterations that they may induce.

To date, stable isotope studies on host-parasite interactions have rarely included comparisons with uninfected conspecifics from the same ecosystem [4, 38], nor have they accounted for temporal variation or different developmental stages of parasites. In this study, we therefore analyzed the trophic positions of uninfected, acanthocephalan-infected and microsporidian-infected *A. aquaticus* individuals from a stream in Germany in four different months using SIA. Furthermore, we measured the SI signatures of the cystacanths of *Acanthocephalus* spp. We hypothesize that i) host sizes, prevalences, and isotope signatures exhibit variations according to the time of sampling, attributable to the host’s age and fluctuations in food availability, ii) the development of acanthocephalans and microsporidians differentially affects host size, metabolism, and nutrient assimilation, with the latter being reflected by shifts in δ^13^C and δ^15^N values, and iii) cystacanths have comparable or lower δ^15^N values than their hosts due to passive absorption of nutrients.

## Methods

### Sampling

We collected individuals of *A. aquaticus* from the brook Oelbach (Bochum, western Germany; 51.438109, 7.283023), a tributary that discharges into Lake Kemnade, which is a reservoir lake of the Ruhr River. In the upstream direction from the sampling location, the Oelbach receives effluents from a wastewater treatment plant located approx. 1.5 km away. Samplings took place in September and November 2023, December 2024, and January 2025, covering three different seasons. The water parameters pH, conductivity, and temperature were measured at each sampling (see Table S1). We collected isopods using household sieves and soft forceps. For transport to the laboratory, they were kept in a 10 L-bucket filled with water and leaves from the site; a battery-driven pump and an airstone were used for aeration. Animals were kept alive in the laboratory until dissection (max. three days). To reduce thermal stress, individuals collected on colder days (approx. ≤ 10 °C) were kept in a refrigerator (approx. 8 °C), otherwise at room temperature (approx. 20 °C).

### Dissection of isopods

We measured the pleotelson widths of *A. aquaticus* individuals as an index for body size (adapted from Kakizaki *et al.* 2003) under a binocular with an attached camera and corresponding camera software (ZEN-lite NT6.2.9200.0; WaveImage 4.11.20351). Owing to a recording oversight, the pleotelson width was not taken for two uninfected and one Acanthocephala-infected isopod (Table S2). Individuals were divided in the transversal plane to remove acanthocephalan cystacanths that were positioned centrally in the haemocoel. Cystacanths were placed in distilled water to induce proboscis eversion for morphological identification; however, complete eversion was not achieved in all individuals, preventing identification in some cases. Therefore, we took small tissue samples from the metasoma of each individual for molecular identification, while using the remaining tissue for SIA. Isopods were sagittally sectioned to remove the intestinal tract and prevent contamination from gut contents. One body half was preserved in 96% ethanol at −20 °C for DNA extraction, the other half was frozen at −20 °C for SIA. We decontaminated scalpels and forceps with 2% bleach and distilled water before each dissection.

### Molecular identification

**Figure S1:**
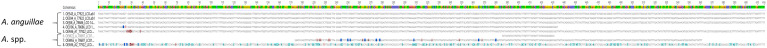
Multiple sequence alignment for Acanthocephala parasitizing the isopod *Asellus aquaticus*. This figure was generated in Geneious (Biomatters).

**Figure S2:**
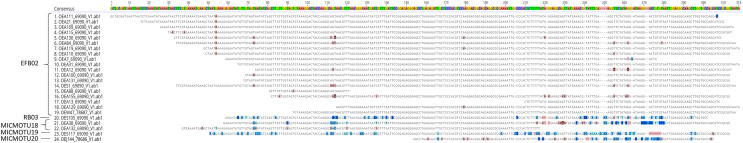
Multiple sequence alignment for Microsporidia parasitizing the isopod *Asellus aquaticus.* This figure was generated in Geneious (Biomatters).

The DNA extraction of isopod and acanthocephalan tissues followed the salt precipitation protocol described in Grabner *et al.* [22] to molecularly identify parasites and hosts (for primer details and PCR conditions, see Table 1). A subset of the sampled isopods from each sampling event (*n* = 113; Table S2) was barcoded with LCO1490/HCO2198, a primer pair designed to target a wide spectrum of invertebrates [17], to verify that the assessed isopod population consists of *A. aquaticus*. We used the same primer pair to barcode acanthocephalans. DNA extracts of isopods and acanthocephalans were tested for microsporidian infections using V1F/micuni3R, a primer pair that has already been used to detect Microsporidia in *A. aquaticus* and that can target a broad range of classical microsporidians [13, 20]. PCR products of positive samples were sent for Sanger sequencing (Microsynth Seqlab, Göttingen, Germany) along with the respective forward primer. Resulting DNA sequences were checked and corrected in Geneious (v.2024.0.3, Biomatters Ltd., Auckland, New Zealand), and aligned against the NCBI database (https://www.ncbi.nlm.nih.gov). The pairwise identity threshold for identification was 98%. Multiple sequence alignments of acanthocephalan and microsporidian sequences were performed in Geneious using the Geneious alignment function. We based our final species assignment on these alignments (Figs. S1, S2), as this approach allowed us to verify the identification of some shorter sequences under 100 bp as well. As it was not possible to generate nucleotide sequences from all acanthocephalan DNA extracts, we grouped all specimens as *Acanthocephalus* spp. for statistical analyses, based on their overall morphology and common intermediate host. Novel microsporidian isolates were named in accordance with Grabner *et al.* [20], starting with the nomenclature “MICMOTU18”. Novel DNA sequences from our study were deposited in GenBank under accession numbers PX113177–PX113180. For MICMOTU18, a consensus sequence was generated in Geneious and uploaded to NCBI.

**Table 1:** Primers and PCR conditions used in this study to barcode two parasite groups (Acanthocephala, Microsporidia) and their isopod host A. aquaticus.

Primers (5′–…–3′)	Target	Primer reference	PCR program
V1F	Classical Microsporidia; partial 18S SSU rRNA gene	Zhu *et al.* [72]; Weigand *et al.* [67]	94 °C, 3 min;40× (94 °C, 35 s; 68 °C, 40 s); 68 °C, 5 min
CACCAGGTTGATT			
CTGCCTGAC			
micuni3R			
ATTACCGCGGMTG			
CTGGCAC			
LCO1490	Metazoan invertebrates; Partial CO1 gene	Folmer *et al.* [17]	94 °C, 3 min; 40× (94 °C, 40 s;58 °C, 40 s;65 °C, 50 s);30× (94 °C, 40 s;53 °C, 30 s;65 °C, 50 s);72 °C, 5 min
GGTCAACAAATCA			
TAAAGATATTGG			
HCO2198			
TAAACTTCAGGGTG			
ACCAAAAAATCA			

### Stable isotope analysis

After dissection, acanthocephalans and isopods were freeze-dried, homogenized and folded into 4 × 6 mm tin capsules (IVA Analysentechnik e.K., Meerbusch, Germany). For each sampling month, we considered 17–33 uninfected isopods, all infected individuals as well as all cystacanths for the SIA (Table S2). The SI compositions of carbon and nitrogen in the selected acanthocephalan and isopod samples were analyzed using an isotope ratio mass spectrometer (IRMS, Isoprime visION, Elementar, Germany) connected to an elemental analyzer (EA, Vario ISOTOPE Select, Elementar, Germany) operating in CN-mode. The isotope ratios δ^13^C and δ^15^N were calculated and reported in δ-notation as differences of the isotope ratio of the sample and isotope ratio of an international reference substance (for details, see [37]). The measurements were performed as triplicates if sufficient sample material was available. However, smaller isopods and acanthocephalans were analyzed as single measurements or several samples were pooled to reach the required minimum mass for analysis. It is also noted that one acanthocephalan in November 2023 was not analyzed as its dry mass was too low. Acetanilide was used as a laboratory internal standard and was normalized using the international standards USGS40 and USGS41a (both International Atomic Energy Agency, Vienna). The measured values of replicates were summarized to means and standard deviations to represent the respective sample in further analyses.

### Data analyses

Data analyses and figures were prepared in R v.4.3.3 [53] implemented in RStudio v.2023.09.0+463 [46], including the packages ggpubr v.0.6.0 [28], ggtext v.0.1.2 [70], RColorBrewer v.1.1-3 [40], readxl v.1.4.3 [69], tidyverse v.2.0.0 [68], and wesanderson v.0.3.7 [54]. The prevalence of infection (*P*%) was calculated for each parasite group (Acanthocephala, Microsporidia) according to Bush *et al.* [7]. We generally focused our analysis on uninfected and acanthocephalan-infected isopods as well as on isopods infected with the microsporidian isolate EFB02, because these groups had sufficient sample sizes in our data set. Accordingly, groups with fewer than three infected individuals (*i.e.*, the microsporidian isolates RB03 and MICMOTU18-20) were only briefly mentioned. It is noted that we combined the two samplings in November 2023 in our analyses (see Table S1). To test the effect of infection on pleotelson width, we used the Wilcoxon test and compared the sizes between the aforementioned groups for each sampling time. September 2023 was not considered in this comparison due to a low sample size of infected individuals (*i.e.*, two acanthocephalan-infected isopods and three microsporidian-infected isopods with different microsporidian isolates each). In order to identify possible differences in diet or trophic level of the isopods in relation to infection status and in comparison with the cystacanths of *Acanthocephalus* spp., differences (Δδ) in mean δ^13^C and δ^15^N values between the respective groups were calculated and compared for each sampling time.

## Results

### Prevalences

In total, we sampled 538 isopods, with about 150 individuals for each sampling month besides December 2024, where only 80 individuals could be retrieved (Table 2, Table S1). For a subset of these isopods, we retrieved 112 DNA sequences that identified them as *Asellus aquaticus* (99.4–100% pairwise identity, Table S2), and therefore we considered all isopods in this study to be *A. aquaticus*.

**Table 2:** Number of isopods, and their infections with acanthocephalans and microsporidians.

Sampling	Isopods	Acanthocephala-infected (prevalences)	Microsporidia-infected (prevalences)
September 2023	153	2 (1.31%)	3 (1.96%)
November 2023	155	3 (1.94%)*	19 (12.26%)
December 2024	80	4 (5.00%)	1 (1.25%)
January 2025	150	6 (4.00%)	1 (0.67%)
In total:	538	15 (2.79%)	24 (4.46%)

* One host was infected with two acanthocephalans in November 2023.

**Figure 1:**
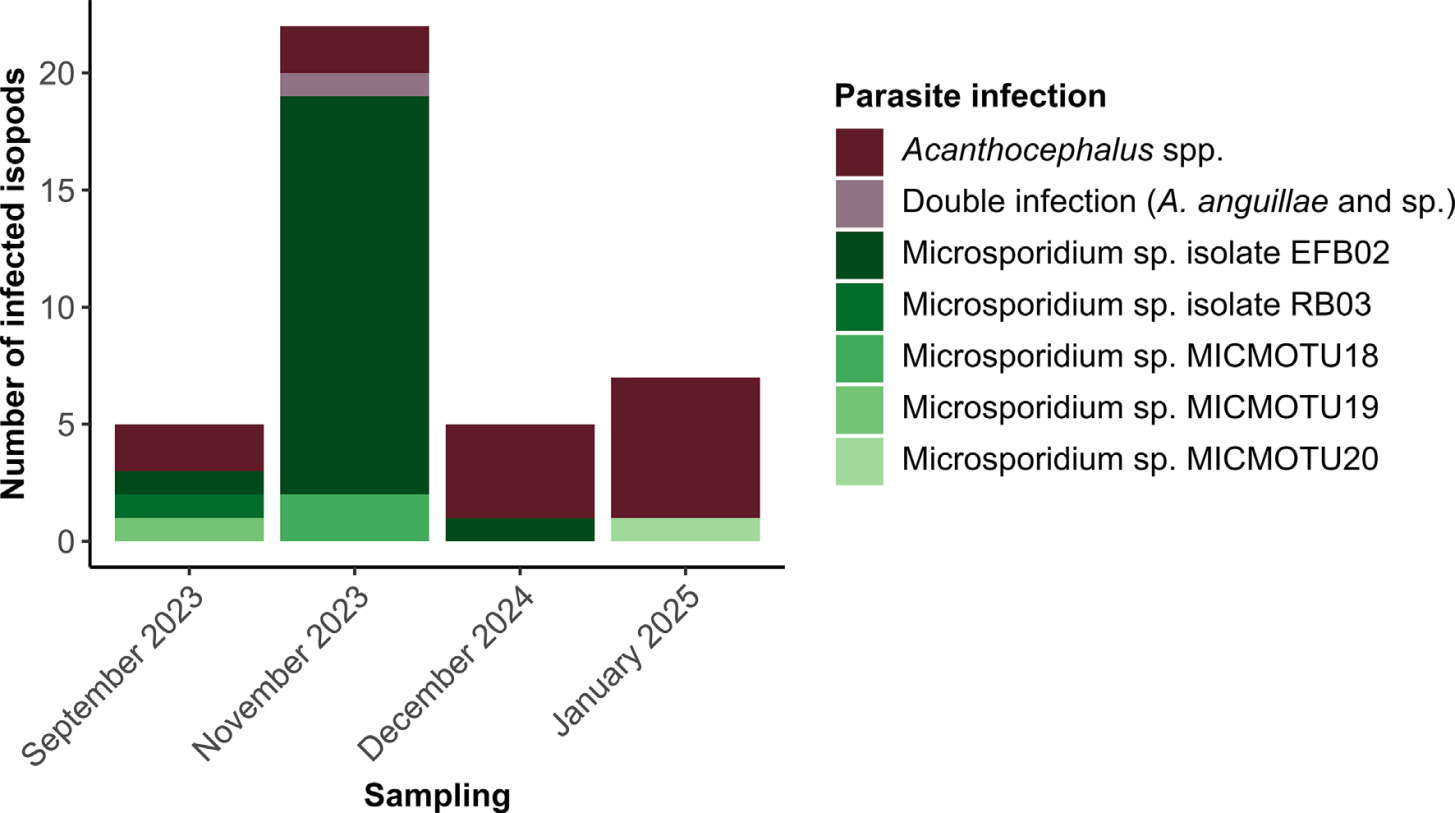
Number of *Asellus aquaticus* individuals infected with acanthocephalans or microsporidians sampled in September 2023 (*n =* 153), November 2023 (*n =* 155), December 2024 (*n =* 80), and January 2025 (*n =* 150).

In total, 15 isopods were infected with one acanthocephalan each, whereas one individual had a double infection with two cystacanths (Fig. 1, Table 2). Most infected isopods were found in December 2024 (*n* = 4, *P* = 5%) and January 2025 (*n* = 6, *P* = 4%), and the fewest in September 2023 (*n* = 2, *P* = 1.3%). We molecularly identified five cystacanths as *Acanthocephalus anguillae* and two as *Acanthocephalus* sp. (98.8–99.5% and 98.3% pairwise identities; Table S2). Of note, the double infection included two different species, *A. anguillae* and *Acanthocephalus* sp. (Table S2). For the remaining nine individuals, we yielded either no or too short DNA sequences and thus could only assign them to the genus *Acanthocephalus*.

We detected microsporidian infections in 24 isopods (Fig. 1, Table 2). The prevalence was highest in November 2023 (*n* = 19, *P* = 12.26%), and lowest in January 2025 (*n* = 1, *P* = 0.67%). The most frequent assignment was Microsporidium sp. EFB02 (*n* = 19, 98.3%–100% pairwise identity, Table S2). We also found microsporidian isolate RB03 in one individual from the September 2023 sampling (99.8% pairwise identity; Table S2). Moreover, we identified three novel microsporidian isolates, named MICMOTU18, −19, and −20. Two isopods were infected with MICMOTU18 in November 2023, while MICMOTU19 and MICMOTU20 were single findings, the former in September 2023, and the latter in January 2025 (Fig. 1, Table S2).

No mixed infections with acanthocephalans and microsporidians were detected, nor was microsporidian hyperparasitism observed in acanthocephalans.

### Host size

**Figure 2:**
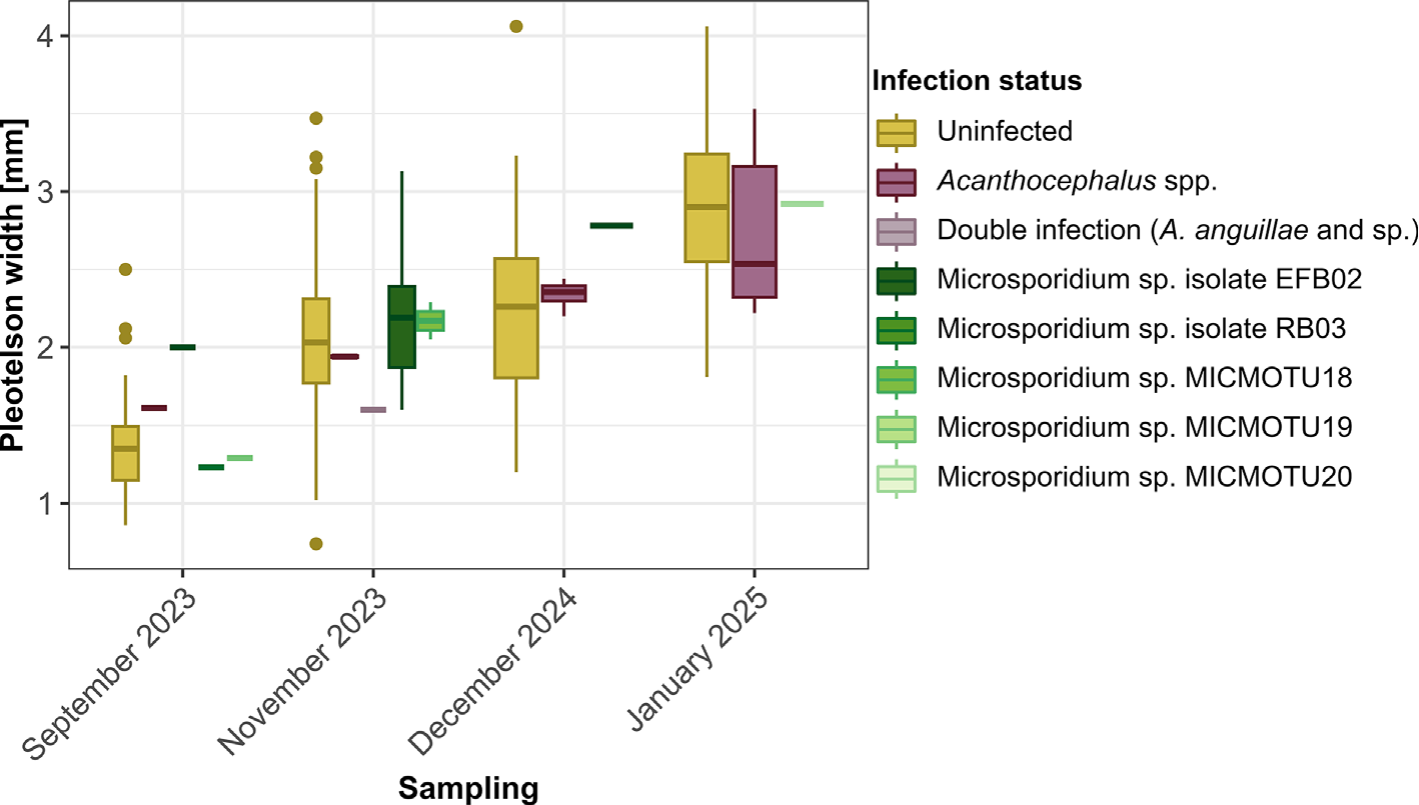
Pleotelson widths of the assessed *A. aquaticus* individuals (*n =* 535), according to sampling and infection status.

**Figure S3:**
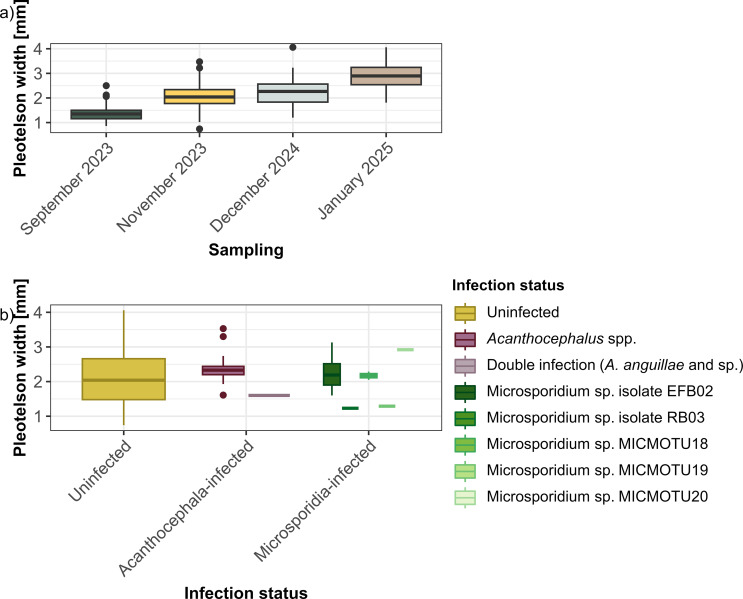
Pleotelson widths according to a) sampling month, and b) infection status.

The pleotelson width of *A. aquaticus* varied between *ca.* 0.74 and 4.06 mm, and was 2.11 mm on average (Figs. 2 and S3; Table S3). The smallest isopods were found in September 2023 (mean: 1.36 ± 0.26 mm) and the largest in January 2025 (mean: 2.88 ± 0.47 mm), whereas the size of isopods from the autumn samplings, November 2023 and December 2024, ranged in between (mean in 2023: 2.07 ± 0.44 mm, mean in 2024: 2.23 ± 0.52 mm). Isopods infected with the microsporidian isolate EFB02 tended to be larger than uninfected isopods, while the individual infected with two acanthocephalans was smaller than roughly 87% of all isopods from the same sampling (Fig. 2, Table S3). Nevertheless, the infection status had no significant effect on pleotelson widths (all *p*-values > 0.05, Table S4).

**Figure 3:**
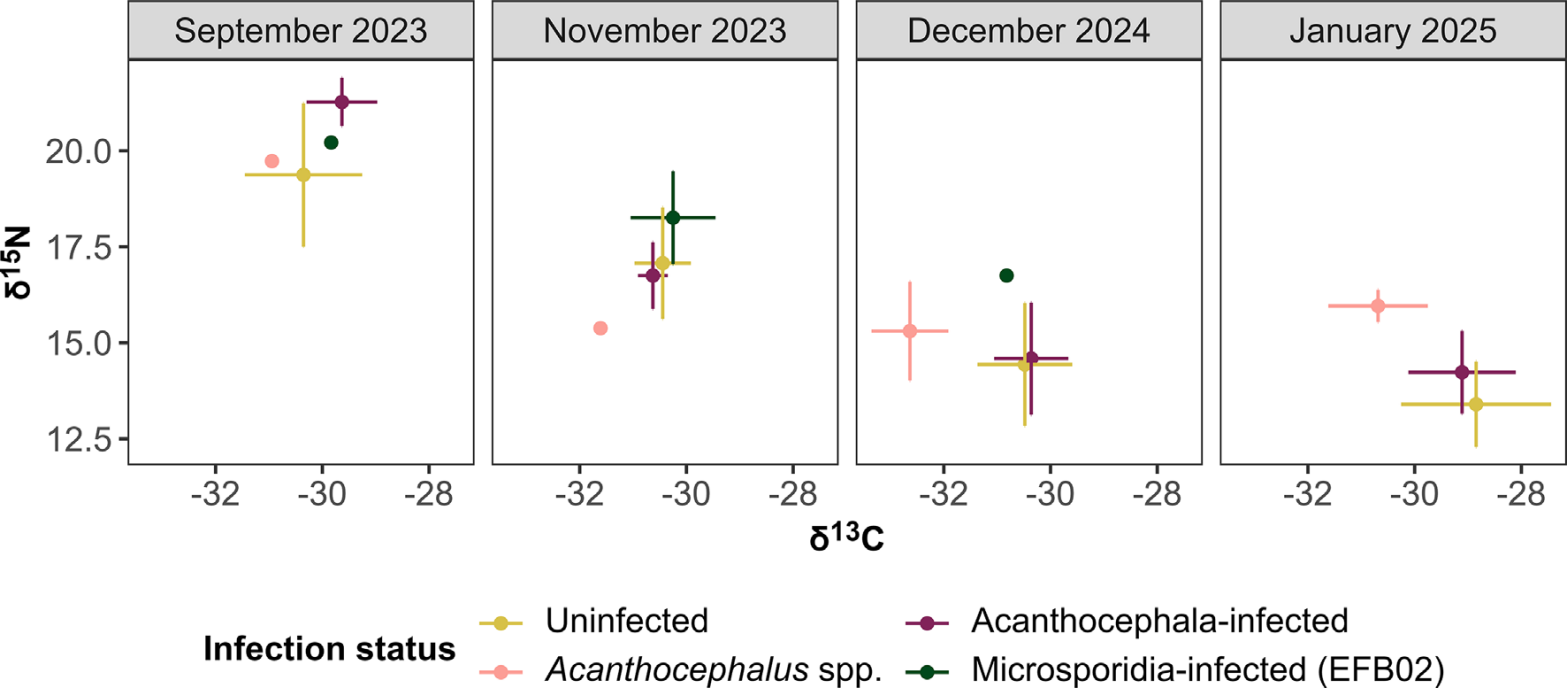
Means and standard deviations of δ^13^C and δ^15^N values of isopods and cystacanths (*Acanthocephalus* spp.). Isopods were further differentiated according to their infection status. Only the most abundant isolate was considered for the group of microsporidian-infected isopods (isolate EFB02). While acanthocephalan-infected isopods were measured individually, acanthocephalans were pooled in September and November 2023. In addition, one acanthocephalan in November 2023 did not yield enough dry mass for SIA.

**Table 3:** Differences of δ^13^C means and δ^15^N means according to infection status in each sampling month. The comparisons include uninfected isopods, infected isopods (with *Acanthocephalus* spp. or the microsporidian isolate EFB02), and cystacanths of *Acanthocephalus* spp. Other microsporidians found in our study were not considered here due to low sample sizes. Values are reported in ‰.

Sampling	Comparison	Δδ^13^C	Δδ^15^N
September 2023	Uninfected isopods – Acanthocephala-infected isopods	−0.72	−1.90
	Uninfected isopods – Microsporidium sp. EFB02-infected isopods	−0.52	−0.84
	Uninfected isopods – *Acanthocephalus* spp. cystacanths	0.59	−0.36
	Acanthocephala-infected isopods – Microsporidium sp. EFB02-infected isopods	0.20	1.06
	Acanthocephala-infected isopods – *Acanthocephalus* spp. cystacanths	1.31	1.54
	Microsporidium sp. EFB02-infected isopods – *Acanthocephalus* spp. cystacanths	1.11	0.48
November 2023	Uninfected isopods – Acanthocephala-infected isopods	0.18	0.32
	Uninfected isopods – Microsporidium sp. EFB02-infected isopods	−0.19	−1.18
	Uninfected isopods – *Acanthocephalus* spp. cystacanths	1.17	1.69
	Acanthocephala-infected isopods – Microsporidium sp. EFB02-infected isopods	−0.25	−1.55
	Acanthocephala-infected isopods – *Acanthocephalus* spp. cystacanths	0.98	1.37
	Microsporidium sp. EFB02-infected isopods – *Acanthocephalus* spp. cystacanths	1.36	2.88
December 2024	Uninfected isopods – Acanthocephala-infected isopods	−0.12	−0.15
	Uninfected isopods – Microsporidium sp. EFB02-infected isopods	0.34	−2.32
	Uninfected isopods – *Acanthocephalus* spp. cystacanths	2.15	−0.87
	Acanthocephala-infected isopods – Microsporidium sp. EFB02-infected isopods	0.46	−2.16
	Acanthocephala-infected isopods – *Acanthocephalus* spp. cystacanths	2.27	−0.72
	Microsporidium sp. EFB02-infected isopods – *Acanthocephalus* spp. cystacanths	1.81	1.45
January 2025	Uninfected isopods – Acanthocephala-infected isopods	0.26	−0.83
	Uninfected isopods – *Acanthocephalus* spp. cystacanths	1.84	−2.56
	Acanthocephala-infected isopods – *Acanthocephalus* spp. cystacanths	1.57	−1.73

### Stable isotope analysis

**Figure S4:**
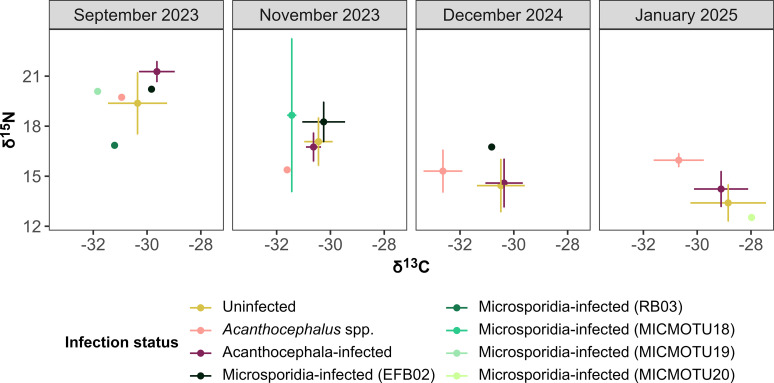
Means and standard deviations of δ^13^C and δ^15^N values of isopods and cystacanths (*Acanthocephalus* spp.). Isopods were further differentiated according to their infection status. Microsporidian single findings are included here. While acanthocephalan-infected isopods were measured individually, acanthocephalans were pooled in September and November 2023. In addition, one acanthocephalan in November 2023 did not yield enough dry mass for SIA.

We analyzed acanthocephalans, tissue of parasitized isopods, and a subset of uninfected isopods from each sampling month (Table S5). As the microsporidian isolate EFB02 was the most prevalent one among all microsporidians, we focused our SI analyses on the differences between uninfected isopods and those infected with EFB02, as well as acanthocephalans and their respective hosts. Data for rare microsporidian isolates (*i.e.*, RB03 and MICMOTU18–20) can be found in the supporting information (Fig. S4, Table S5).

The SI values of δ^13^C and δ^15^N varied across sampling months and between infection statuses (Fig. 3). In September 2023, Acanthocephala-infected *A. aquaticus* exhibited higher δ^15^N values compared to both, the uninfected and microsporidian EFB02-infected isopods (Δδ^15^N = 1.90‰ and 1.06‰, respectively; Fig. 3, Table 3). However, in November 2023 and December 2024, the δ^15^N values of Acanthocephala-infected individuals were similar to those of uninfected isopods. In January 2025, δ^15^N of Acanthocephala-infected isopods again exceeded those of uninfected ones (Δδ^15^N = 0.83‰). In September 2023, Acanthocephala-infected isopods showed higher δ^13^C values than those of uninfected ones (Fig. 3, Table 3). In contrast, δ^13^C values in Acanthocephala-infected isopods were comparable to uninfected isopods in both autumn samplings November 2023 and December 2024. In January 2025, δ^13^C levels of Acanthocephala-infected isopods were lower than those of the uninfected isopods.

*Acanthocephalus* spp. cystacanths showed lower δ^15^N than all other groups in November 2023. In January 2025, however, the δ^15^N values were higher than those of uninfected or Acanthocephala-infected isopods (Δδ^15^N = 2.56 and 1.73%, respectively; Figure 3, Table 3). In September 2023 and December 2024, the δ^15^N values were similar to those of the uninfected isopods. Across all sampling months, δ^13^C values in this group remained relatively stable but consistently lower than those of the other groups.

Microsporidian EFB02-infected isopods showed elevated δ^15^N values compared to uninfected isopods and *Acanthocephalus* spp. cystacanths. The δ^13^C values in EFB02-infected isopods were generally consistent across sampling months and similar to those of uninfected individuals (Figure 3). A comparison according to the different samplings was not possible, as November 2023 was the only sampling event with more than one individual infected with this microsporidian isolate.

## Discussion

In the present study, we analyzed the parasite community of Acanthocephala and Microsporidia in *A. aquaticus* and studied the isotope profiles of cystacanths as well as of infected and uninfected hosts to identify potential parasite-induced changes in the hosts’ trophic ecology. To detect parasite-induced alterations, host resource availability and temporal dynamics must be considered. *Asellus aquaticus* consumes a wide range of food sources, including algae, bacteria, detritus, fungi, macrophytes, and associated periphyton [23, 33, 36, 65, 71], with the food supply typically changing during the year and being highest in spring and summer [58]. Additionally, the feeding activity and metabolic rates of isopods, as poikilothermic organisms, play an essential role that might shape their SI patterns as well. The SI signatures in our study gradually differed between the sampling months, indicating that *A. aquaticus* likely underwent dietary changes during the year. From September 2023 (late summer) to January 2025 (winter), the isopods appeared to switch to a more plant-based diet, as ^13^C enrichment indicates more vegetation and ^15^N depletion indicates shorter food chains [34, 47]. Observations of uninfected *A. aquaticus* individuals served as the baseline for analyzing the two host-parasite systems: *Asellus aquaticus-Acanthocephalus* spp. and *A. aquaticus*-Microsporidium EFB02.

Although we summarized all acanthocephalans as *Acanthocephalus* spp., we were able to identify two different acanthocephalans in total molecularly. Those acanthocephalans that yielded nucleotide sequences were *A. anguillae* and *Acanthocephalus* sp. [55]. Importantly, in this case, the taxonomic assignment “*Acanthocephalus* sp.” refers to an actual genetically distinct lineage whose sequence is deposited in the NCBI database (accession no. MT682935). Based on sequence divergence, an assignment to other *Acanthocephalus* species such as *A. ranae* that uses amphibian hosts can therefore be excluded. The prevalences of all of these *Acanthocephalus* spp. cystacanths taken together showed a temporal pattern with the highest prevalence in December 2024 (late autumn) and January 2025 (winter), and the lowest in September (late summer) and November (autumn) 2023. Temporal patterns in prevalences in intermediate and final hosts are a known phenomenon in acanthocephalans, for example in *Pomphorhynchus laevis* [39]. This might be explained, at least in part, by the life cycle of these parasites. For example, cystacanths manipulate their isopod intermediate hosts during the winter and spring months by increasing their activity and thus their susceptibility to predation by the fish final hosts ([2] and references therein). This behavior may also have contributed to a higher frequency of infected isopods being caught, as evidenced by the higher prevalence in December 2024 and January 2025. Conversely, isopods likely just start to become infected with acanthors during the summer months ([2] and references therein). A lower prevalence in September 2023 compared to later months can further depend on the life cycle of *A. aquaticus*, as overwintered and thus older cohorts may have died after the breeding season, thereby introducing a new isopod generation that is therefore not yet infected with acanthocephalan larvae [5].

The SI patterns obtained for *A. aquaticus*-*Acanthocephalus* spp. might further reflect the seasonality in the life cycle of *Acanthocephalus* species, starting with the time at which the parasite infects its intermediate host and followed by a phase of growth and development during the year. *Asellus aquaticus* likely gets infected especially during summer by ingesting eggs containing acanthor larvae [5, 6]. The subsequent growth and development of these larvae is known to correlate with higher water temperatures [5, 63]. If the development into infective cystacanths is not completed until autumn, development is arrested at colder temperatures and larvae reach the cystacanth stage in spring, as it was shown for *A. lucii* and *A. anguillae* [5, 63]. Therefore, the cystacanths found in September 2023 could result from infections in the previous year. In either scenario, the *Acanthocephalus* spp. larvae are likely energy-demanding for their host around summer, causing higher δ^15^N signatures in isopods as a sign of starvation or due to dietary changes [4, 37, 45]. It is noteworthy that the δ^15^N values measured in the cystacanths were on average higher than those of their isopod hosts in December 2024 and January 2024. The isotopic enrichment may be indicative of their elevated trophic position relative to the host tissue, potentially due to selective assimilation of host nutrients or metabolic fractionation during development [25, 38]. This result contrasts previous findings on adult Acanthocephala, which were depleted in ^15^N compared to their hosts (*e.g.* [37], and reviewed in [38]), indicating that the developmental stage might be a relevant factor. Changes in isotope ratios during ontogeny have already been described in a parasitic crustacean [19] and may also occur for other metazoan parasites such as acanthocephalans.

Among the five microsporidian isolates detected in the present study, two are already known from German streams in North Rhine-Westphalia: the most prevalent isolate, EFB02, was previously detected in one *A. aquaticus* individual from the Finkelbach stream [49, 50], while it was not found in a study on microsporidians in *A. aquaticus* from all over Europe [20]. Isolate RB03 was a single finding in *Gammarus pulex* from the Rotbach stream [48], indicating that RB03 may have low host specificity and infects both amphipods and isopods. The remaining three microsporidian isolates are, to our knowledge, new findings, pointing towards how much of the microsporidian diversity might still be unknown. Microsporidian prevalences exhibited a different temporal pattern to that of acanthocephalans, with the highest prevalence in November 2023 and the lowest in January 2025. This pattern was mainly driven by the most common microsporidian isolate EFB02. Little is known about this isolate, including the factors that may lead to the observed varying prevalences. Possible explanations could again be found in the host’s life cycle. Besides the aforementioned possible occurrence of a new host generation around summer (*e.g.* [5]), some *A. aquaticus* populations can undergo reproductive diapauses (*e.g.* [66]), which may be a relevant factor for microsporidians using vertical transmission pathways. In addition, a varying host density can affect the probability of transmission (*e.g.* [16]). Environmental factors like temperature differences are also important, as microsporidian transmission and burden can be impaired at low temperatures (*e.g.* [15, 16]).

Despite finding five different microsporidian isolates, we only considered isolate EFB02 for our SIA analyses due to its higher prevalence. In contrast to acanthocephalans, microsporidian infections in *A. aquaticus* exhibited distinct effects. In the *A. aquaticus*–Microsporidium EFB02 system, infected isopods showed higher δ^15^N values compared to uninfected individuals. Similar ^15^N and ^13^C enrichments have been reported for *Daphnia* sp. infected with microsporidians, along with reduced growth and lipid content, symptoms resembling food limitation [52]. The absence of ^13^C shifts or growth reduction in EFB02-infected isopods may reflect different infection timing, tissue tropism, or parasite strategies, highlighting the variability in host manipulation by microsporidians. Although ^15^N enrichment could result from parasite tissue contributions [12], this effect is likely minimal due to the low parasite biomass. Nonetheless, future studies should account for infection intensity and site.

Besides our expectations to uncover alterations in the trophic ecology of *A. aquaticus* induced by acanthocephalans and microsporidians, we assumed that both groups may also impact other host traits, including body size. For acanthocephalans, previous studies on isopods reported that individuals infected with acanthocephalans can become larger than uninfected ones (*e.g.* [27]). However, we did not observe significant size differences, possibly due to the low prevalence. For microsporidians, size reductions have been described, as in the aforementioned study on microsporidian-infected *Daphnia* [52], but also that hosts can become larger than their uninfected conspecifics (*e.g.* [20])*.* In our study, we found non-significant trends for the hosts of the microsporidian isolate EFB02, which indeed tended to be larger than uninfected ones. This tendency is supported by similar observations described by Grabner *et al.* [20], according to whom, microsporidian-infected *A. aquaticus* were generally larger than uninfected individuals. Some Microsporidia have the ability to hamper their hosts’ sexual maturation, resulting in longer growth phases before reaching maturity (*e.g.* [14]). However, the infected isopods could also be older and thus larger, thereby indicating a horizontal transmission mode of EFB02, as such infections typically accumulate over time. Subsequent studies should assess morphological changes induced by this microsporidian, also under consideration of sexual dimorphism or sex ratios, as has already been done for other microsporidian-susceptible crustaceans like gammarids (*e.g.* [64]).

Sample sizes were limited in some months, so findings on host size and trophic positions of parasitized isopods and acanthocephalans should be interpreted with caution. Additionally, treated wastewater input into the stream may have influenced isotopic signatures. To minimize this effect, we focused on within-month comparisons (infected *vs.* uninfected, host *vs.* cystacanth) rather than between-month patterns. Wastewater is typically enriched in ^15^N due to microbial processing, which can elevate ^15^N in downstream organisms [35, 56], likely contributing to the generally high nitrogen values observed in our study.

## Conclusion

Taken together, our study contributes to the characterization of two parasite groups in the keystone species *A. aquaticus* and especially highlights the distinct influence of Acanthocephala and Microsporidia on the trophic ecology of their host. We detected infections with *Acanthocephalus* spp. and with five different microsporidian isolates, three of which, to our knowledge, were previously unknown. Hypothesis 1 was generally supported by our findings, as we identified differences in host size, prevalences and SI signatures between the sampling events. Our expectations regarding parasite-induced alterations in *A. aquaticus* as defined by hypothesis 2 was partially met: Despite a tendency of microsporidian EFB02-infected hosts being larger than uninfected conspecifics, no significant size differences were detected between uninfected and infected isopods. However, the SIA demonstrated that an isopod’s trophic signature can be altered by acanthocephalans and microsporidians, reflecting changes in metabolism and diet that could consequently influence host fitness and ecosystem nutrient cycling, and that these alterations can differ between these two parasite groups. Hypothesis 3, assuming lower δ^15^N values in cystacanths than in their hosts, was evident especially in September and in November 2023. In the subsequent sampling events, the cystacanths’ δ^15^N stayed on a similar level as in November 2023, causing stronger differences between cystacanths and hosts, with higher levels in the cystacanths. We thus conclude that their nutrient uptake remained stable, while food sources for their hosts changed. This study contributes to our understanding of the role parasites can play in trophic ecology, and emphasizes the necessity of integrating parasitological, ecological, and environmental perspectives to comprehensively understand parasite impacts in natural systems.

## Supplementary material

*Table S1*: Water parameters and number of collected isopods for each sampling. *Table S2*: Overview of host individuals, acanthocephalans, and microsporidians. *Table S3*: Minimum (min), maximum (max), mean and standard deviation (sd) of pleotelson widths of *A. aquaticus*, as well as the number of measured individuals (n), according to sampling month and infection status. Values rounded to two decimals. Note that the total number of measured isopods is 535, as three pleotelson widths were not taken. *Table S4*: Pairwise comparisons of the pleotelson width of *A. aquaticus* between different infection statuses, according to sampling time. *P*-values and corresponding significance levels are indicated as follows: *p* < 0.05 = *, *p* < 0.010 = **, *p* < 0.001 = ***, ns = not significant. *Table S5*: Measured δ^15^N and δ^13^C values in isopods and acanthocephalans. “Mean” columns include the mean values of replicates as well as individual measurements if no replicates were available. “SD” columns include the corresponding standard deviation if samples had replicates. The supplementary material of this article is available at https://www.parasite-journal.org/10.1051/parasite/2025063/olm.
